# Treatment of Tetanus

**Published:** 1890-02

**Authors:** 


The Treatment of Tetanus.—We are told by *Chambers' Journal* that Prof. Renzi, of Naples, has treated successfully several cases of tetanus, by absolute rest for the patient. This absolute rest does not mean simple release from labor, but includes rest for the several senses, as well as for the body. The ears of the patient are closed with wax, the room is darkened, and the floor is heavily carpeted. Every fifteen minutes the nurse enters with a shaded lantern to attend to his wants, and to administer food, such as eggs, milk, and other fluids. Nothing solid or requiring any attempt to masticate is given. Sedatives are administered as required to relieve pain. It is said this treatment shortens but little, if any, the length of the disease, but it lessens the force of the paroxysms, which gradually cease altogether.—*Canadian Practitioner.*

Medical men in general are probably not aware that in France the doctors' claim on the estate of a deceased patient has precedence of all others. Even the landlord's claims for arrears of rent must yield to the doctor's fee. Tho courts have decided that as it is an imperative right of humanity that the dying should have the necessary care and treatment, such attendance should be paid for before all other debts.—*Scientific American.*

